# Computer-assisted evaluation enhances the quantification of interstitial fibrosis in renal implantation biopsies, measures differences between frozen and paraffin sections, and predicts delayed graft function

**DOI:** 10.1007/s40620-022-01315-y

**Published:** 2022-04-19

**Authors:** Mladen Pavlovic, Andre Oszwald, Željko Kikić, Maja Carina Nackenhorst, Renate Kain, Nicolas Kozakowski

**Affiliations:** 1 Department of Pathology, Medical University of Vienna, General Hospital, Waehringer Guertel 18-20, 1090 Vienna, Austria; 2 Department of Urology, Medical University of Vienna, Vienna, Austria; 3 Institut National de la Santé et de la Recherche Médicale (INSERM), UMRS 1155, Tenon Hospital, Paris, France

**Keywords:** Implantation biopsy, Interstitial fibrosis, Computer-assisted evaluation, Delayed graft function

## Abstract

**Background:**

(Pre-)Implantation biopsies provide important data on the quality of donor kidneys. Interstitial fibrosis, as a known predictor for kidney disease progression, is an essential feature of this evaluation. However, the assessment of frozen sections of implantation biopsies is challenging and can result in the disposal of candidate organs. We sought to apply digital image analysis (DIA) to quantify the differences between frozen and paraffin sections when evaluating interstitial fibrosis, identify factors that influence these variations and test the predictive value of the computerised measures.

**Methods:**

We quantified the differences between frozen and paraffin sections in the same biopsy samples by measuring Sirius red-stained interstitial areas (SRIA) in DIA. We compared them to the original reports, and retrospectively correlated our findings to clinical data, graft function and outcome in 73 patients.

**Results:**

Frozen sections display a broader interstitial area than paraffin sections, in some cases up to one-third more (mean difference + 7.8%, range − 7 to 29%). No donor-related factors (age or gender, cold ischemia time, or non-heart-beating donor) influenced significantly this difference. Compared to the original assessment of frozen vs paraffin sections in optical microscopy, the DIA of interstitial fibrosis shows a higher consistency (ICC 0.69). Our approach further allows to distinguish SRIA in paraffin sections as an independent predictor for delayed graft function (OR = 1.1; *p* = 0.028).

**Conclusions:**

DIA is superior to and more consistent than routine optic microscopy for interstitial fibrosis evaluation. This method could improve implantation biopsy diagnostics and help to reduce disposal of organs.

**Graphical abstract:**

**Supplementary Information:**

The online version contains supplementary material available at 10.1007/s40620-022-01315-y.

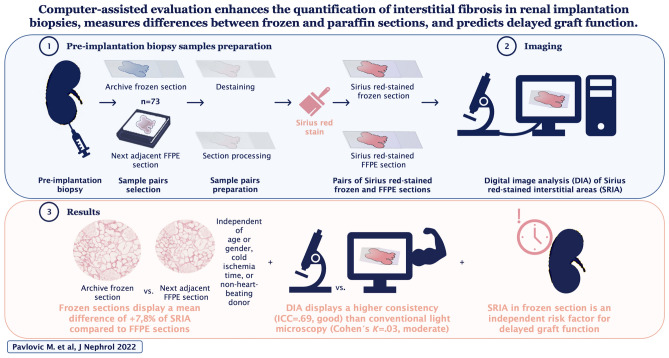

## Introduction

Kidney transplantation is the treatment of choice for end-stage renal disease (ESRD) for social, economic, life quality- and health-related reasons [1]. The demand for grafts is rising, but the current donor pool restricted. These limitations, in turn, increase the time to transplantation and threaten the life of patients affected [2]. The number of transplanted kidneys depends to a certain extent on the organ discard rates that vary mainly due to regional allocation systems and practices. In the US, around 50% of kidneys from donors over 50 years of age are discarded after biopsy assessment made in frozen sections [3], and this percentage is consistently higher than in Europe [4, 5]. Overall, kidneys are significantly more often discarded after biopsy, with an annual discard rate of around 30% compared to less than 10% for organs that had not been biopsied [6]. It is frequently argued that some of these organs might have had a satisfactory function if used [7]. Several studies, however, presented contradictory results, ranging from significant to no association between histologic parameters in (pre-)implantation biopsies and clinical outcome [8–10]. Various factors independent of histology findings, such as recipient-related ones, immunological and environmental influences, may impact on clinical outcome as well, rendering the underlying causes difficult to dissect [11].

One of the most difficult histologic features to assess in a frozen section is the renal interstitium [12–14]. Frozen sections have less stain contrast, and thus, do not always elicit a clear delineation of tissue alterations in comparison to paraffin sections. In addition, the interstitial matrix appears broader and, combined with other artefacts, such as the formation of ice crystals, can lead to an overestimation of interstitial fibrosis [15]. Often a consequence of tubular atrophy, it represents a central element in the assessment of the quality of the organ to be transplanted, as its presence correlates with adverse outcome [16, 17]. Furthermore, fibrosis itself can result in fibrogenesis and inflammation, which in turn could favour alloimmunisation [18]. Histological assessment based on formalin-fixed, paraffin-embedded material is the gold standard for the evaluation of the renal interstitium. This technique is, however, not available immediately before transplantation when a quick evaluation of organ quality is required. Consequently, a reliable measure of the difference in the interstitial area or volume between frozen and paraffin sections would provide critical data for pathologists to adjust fibrosis scores when reporting frozen sections and could lead to strategies for graft preservation, such as an adapted immunosuppression [18, 19].

In addition, the assessment of implantation biopsies in many centres is performed by on-call pathologists who may not regularly report kidney biopsies. Digital pathology, allowing reading slides distant from the laboratory where they are generated by trained nephropathologists, could be used in this case and may improve the quality of pre-implantation assessment [13, 20, 21]. Recent initiatives support this view: the Banff Foundation recently founded the Digital Pathology working group with the objective to enhance the quality of reporting [22], and authors have already applied computerised image analysis to assess fibrosis in native and transplant kidneys [12, 23, 24].

We therefore aimed to quantify the differences in the interstitial compartment assessed in frozen and paraffin sections of donor kidney biopsies at the time of implantation by digital image analysis and explore the factors potentially influencing these variations. Next, we compared the performance of computer-assisted and optical microscopy evaluation of interstitial fibrosis. Finally, we challenged the prognostic value of each method for clinical data and kidney graft outcome.

## Materials and methods

### Biopsy samples

We retrospectively compared paired sections of frozen tissue and the corresponding section after formalin-fixation and paraffin-embedding (paraffin section) using biopsies performed at the time of implantation and archived at the Department of Pathology of the Medical University of Vienna. These biopsies had been obtained as part of the pre-transplantation evaluation of kidney grafts macroscopically (showing scars, reduced size) or clinically (uncontrolled arterial hypertension, diabetes mellitus or proteinuria of the donor) suspicious for chronic renal injury at our centre between 2010 and 2017. All biopsies were evaluated by on-call pathologists, using a score devised by Remuzzi et al. [25]. We performed the study following the Helsinki declaration and with the approval of the local ethics committee (No. 1230/2018).

### Processing

Once received, unfixed, biopsies were snap-frozen, cut in 4-μm thick sections, and stained using haematoxylin and eosin (H&E), after which pre-implantation histological evaluation was performed by on-call pathologists. The tissue was consequently thawed and fixed in 7.5% formalin, embedded in paraffin and cut in 4-μm thick sections for standard nephropathological evaluation by the Department of Pathology’s nephropathology working group. A proportion of 15 biopsies (14.7%) was cut in half whereby one half was used for frozen section with subsequent FFPE processing while the other half was directly fixed and paraffin embedded to evaluate whether prior freezing subsequently influences assessment of the interstitial morphology. To evaluate the interstitial collagen content of the kidney biopsy specimen, the H&E-stained slides from the original frozen section were re-used by soaking them in a xylene substitute to remove the coverslips, followed by immersion in 1% HCl in 80% alcohol for 30 min or until no more residual H&E stain was visible on the section. After a washing step in double-distilled water for 5 min, and 96% and 100% alcohol baths, they were ready for subsequent re-staining. Avoiding post-fixation with formalin of the original frozen section allowed to compare the amount of renal interstitial space in the original frozen section compared to the FFPE section. Sections of FFPE tissue were de-paraffinized, rehydrated in decreasing concentrations of ethanol and all section pairs were stained with Sirius Red, a stain specifically designed to highlight physiological and accumulated collagen [26], and used in several publications for the evaluation of renal interstitial fibrosis in various conditions [27–31]. The sections were left to dry at room temperature for one hour and stained in saturated picric acid with 0.1% Sirius Red F3BA (Sigma-Aldrich, MO, USA). Then the slides were washed in two portions of 30% acetic acid for 2 min, rapidly dehydrated through graded alcohol baths, then n-butyl-acetate, and finally cover-slipped. The sections of the formalin-fixed paraffin-embedded material stained with Sirius Red represented for us the reference morphology and will be considered as such throughout the manuscript.

### Digital evaluation of the sections

**Fig. 1:**
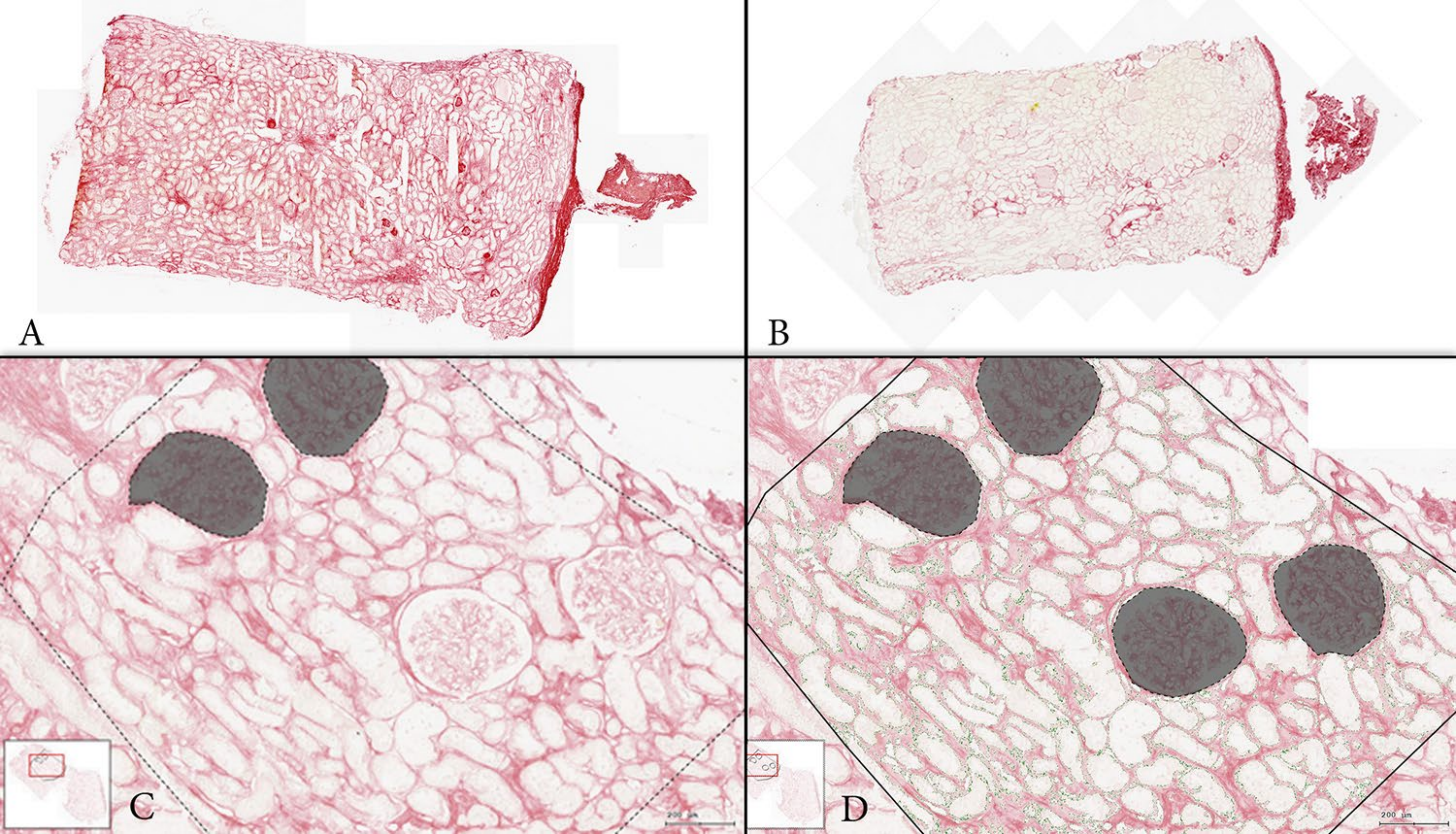
Computer-assisted evaluation of Sirius red stains. **A** Frozen section; **B** paraffin section of the same pair; **C** SRIA in paraffin section before exclusion of glomeruli; **D** SRIA in paraffin section after exclusion of glomeruli

**Fig. 2:**
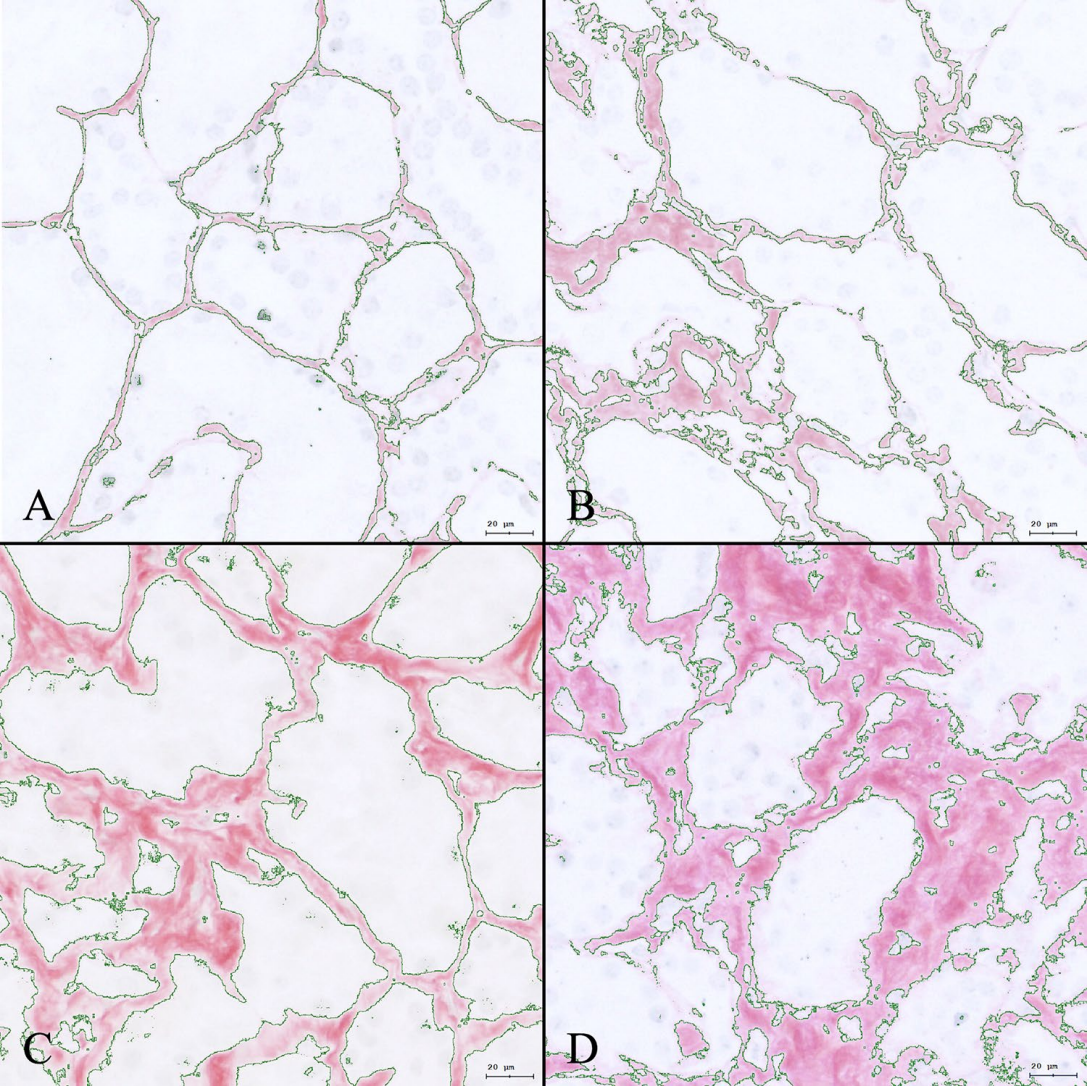
Computer-assisted evaluation of Sirius red stains at higher magnification. **A** Close to no interstitial fibrosis; **B** low-grade interstitial fibrosis; **C** intermediate interstitial fibrosis; **D** high-grade interstitial fibrosis

Using the TissueFAXS SL SlideLoader (TissueGnostics GmbH, Austria) all slides were scanned and whole slide images (WSI) were processed and stored for further digital evaluation with the software TissueQuest (TissueGnostics GmbH, Austria), as previously applied for allograft transplant biopsies [32]. As recommended by the Banff classification of kidney allograft pathology, we exclusively assessed the cortical part of each biopsy specimen [33]. Two experienced nephropathologists from our centre (N.K. and R.K.) manually marked and confirmed the areas of interest before analysis (Fig. 1A, B). Operator-supervised exclusion of technical artefacts, or tissue folds impeding evaluation, and renal corpuscles or prominent blood vessels inside the analysed section area was completed (Fig. 1C, D and higher magnifications in Fig. 2A–D). No interstitial oedema potentially modifying the perception of interstitial fibrosis was detected. To quantify the Sirius red-stained interstitial area (SRIA) based on colorimetric properties, we classified the pixels of each scanned image, based on the automated colour separation into grey-scale images and set a subsequent intensity threshold, performed for each section pair individually. The sections went through a series of testing with different thresholds to obtain the visually most accurate results, and we assumed that the surface area would correlate proportionally with the interstitial volume, as previously described in different tissues, including allograft kidney biopsies [26, 27]. The sample pairs were excluded if the sections or the areas of interest were damaged or if the overlaying area between one frozen and paraffin section pair used for analysis was smaller than 1 mm^2^. The computer-based continuous measurements of interstitial fibrosis expressed in % of the cortical surface was translated into the score proposed by Remuzzi et al. [25] for IF to compare it to the original semi-quantitative evaluation provided by the on-call pathologists.

### Clinical parameters

Baseline data included donor data (age, gender, and donor type: non–heart-beating (NHBD) or brain-dead-donor (BDD)), and recipient data (age, gender, transplantation history, number of HLA mismatches, the peak level of the panel reactive antibody (PRA), the presence of donor-specific antibodies (DSA) and cold ischaemia time (CIT)). Follow-up data with Chronic Kidney Disease Epidemiology Collaboration (CKD-EPI) formula for estimated glomerular filtration rate (eGFR) at 1, 3, 6 and 12 months (M1, M3, M6, and M12, respectively) after transplantation, delayed graft function (DGF, defined as the requirement for dialysis within the first week after transplantation), 1-year rejection episodes and 1-year graft survival completed our records.

### Statistical analysis

We performed statistical analyses and drew graphs using Prism 8.1.1 for MacOS (GraphPad Software, CA, USA), or IBM SPSS Statistics 24 for MacOS (IBM Corporation, Armonk, NY) for tests not available in Prism. Continuous data are expressed as the median and interquartile range (IQR) and categorical variables as absolute and relative frequencies. We performed an unpaired T-test of normally distributed data, alternatively a Mann–Whitney-U-Test for independent samples comparison. We plotted a Bland–Altman diagram, the approach of choice in evaluating two measurement methods and assessing their agreement [34, 35], and tested its model coefficient by linear regression. We calculated the two-way consistency average measure of intraclass correlations (ICCs) for agreement evaluation of continuous measurements and a weighted quadratic Cohen’s Kappa for categorical measurements, to compare the performance of the pathologist- and the computer-based methods. Multiple logistic regression analyses based on factors with p-values < 0.1 in univariate analysis examined the predictive potential of our measurements for the occurrence of DGF or rejection, follow-up eGFR or graft survival. Variance inflation factor tested for collinearity. Significance testing was two-sided, and the results were considered statistically significant at p < 0.05.

## Results

### Study population

In total, we examined 102 implantation biopsy specimens from 85 donors, 17 patients had donated both kidneys in our centre. All corresponding kidneys were subsequently transplanted into 102 recipients. After excluding 29 patients due to missing clinical follow-up data or limitations of sections (see “Materials and methods” section), our study population consisted of 73 patients. The median (IQR) donor and recipient ages were 69 (49–74) and 55 (48–67) years, respectively, with 64.4% male donors and the same percentage of male recipients. The majority of organs (82.2%) originated from BDD, the rest from NHBD. For 12 (16.4%) patients, it was at least the second kidney transplantation. Table 1 summarises the characteristics of our population.

**Table 1:** Study population characteristics

	Number	Total cohort
Donor age (years)*	73	69 (49–74)
Male donor	73	47 (64.4%)
Donor type DBD (vs NHBD)	72	60 (82.2%)
Recipient age (years)*	73	55 (48–67)
Male recipient	73	47 (64.4%)
Re-transplantation	73	12 (16.4%)
HLA mismatches*	69	3 (2–4)
PRA Score > 10%	66	3 (4.1%)
Presence of donor-specific antibodies	73	10 (13.7%)
Cold ischaemia time (hours)*	69	17 (13–22)
Delayed graft function	71	29 (41%)
Serum creatinine at transplantation*	72	0.92 (0.7–1.2)
eGFR at M1 (mL/min/1.73 m^2^)*	69	30.7 (22.95–41.55)
eGFR at M3 (mL/min/1.73 m^2^)*	65	37.2 (30.65–49.55)
eGFR at M6 (mL/min/1.73 m^2^)*	59	35.3 (30.2–50.1)
eGFR at M12(mL/min/1.73 m^2^)*	51	40.8 (33.5–53.5)
One year-graft survival	60	51 (85%)

*BDD* brain-dead donor, *NHBD* non-heart beating donor, *HLA* human leukocyte antigen, *PRA* panel reactive antibody

*Given in median (interquartile range); without * in absolute number (%)

### Frozen section leads to over-estimation of interstitial fibrosis

**Fig. 3:**
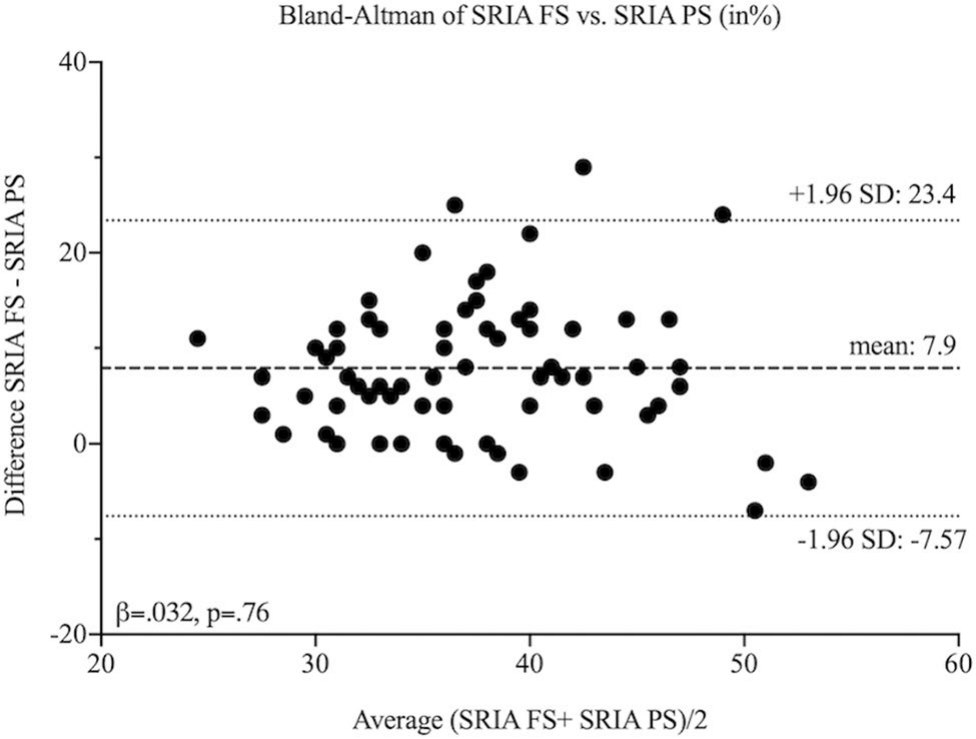
Bland–Altman Plot of SRIA in frozen vs paraffin section (in %). The mean difference between the measurements is 7.9%. The long-dotted line represents the mean difference of SRIA between frozen and paraffin sections and short-dotted lines are the 95% limits of agreement (± 1.96 standard deviation (SD) = − 7.57–23.4%). A linear regression with a β = 0.032 (p = 0.76) demonstrates the absence of proportional bias between the measurement methods. *SD* standard deviation

We obtained mainly core biopsies (98 (96%)), a practice established for several years at our centre [36], and a subset of four wedge biopsies (4%). Median computer-assisted measurements of SRIA in frozen and paraffin sections had a significant median difference of 9% (41% (36–47) and 32% (29–38), respectively, p < 0.001) in favour of frozen sections, whereby we did not observe a significant difference whether the tissue was previously frozen or not. A Bland–Altman plot with a mean difference of 7.8% and a β-coefficient close to zero in linear regression (− 0.039, p = 0.76), showed no proportional bias between the measurements. This meant that frozen sections in computer-assisted measurements did not give gradually higher readings as the percentage of interstitial fibrosis in paraffin sections rose (Fig. 3).

### Computer-assisted assessment of SRIA in frozen and paraffin sections shows higher consistency than the semi-quantitative method

**Fig. 4:**
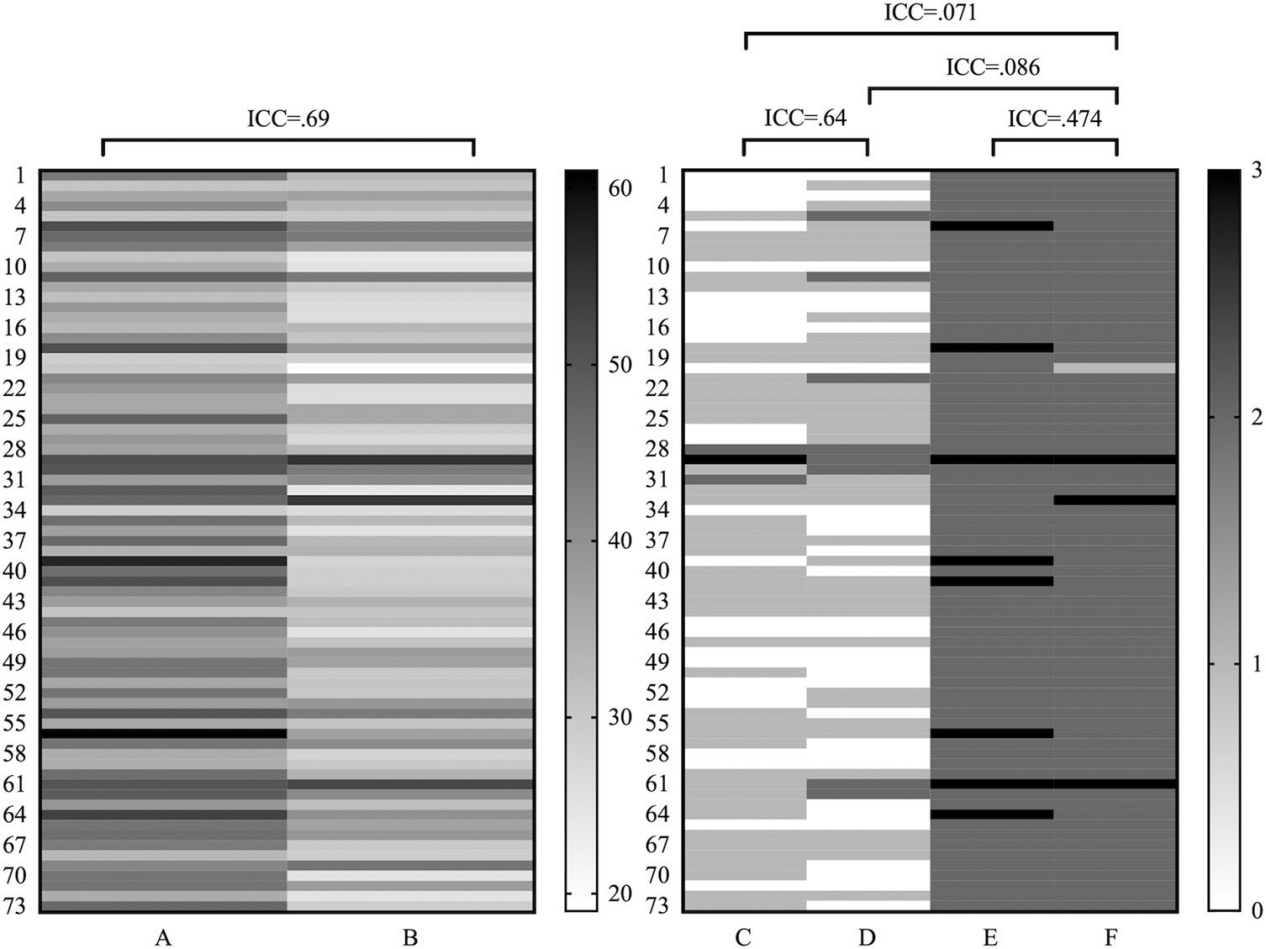
Heat maps of the interstitial fibrosis score according to **A** SRIA in frozen sections; **B** SRIA in paraffin sections; or to Remuzzi et al. [25] performed in **C** frozen section; **D** paraffin section; **E** translated from the SRIA of frozen section; and **F** translated from the SRIA of paraffin sections. While ICC between **A** vs **B** was good, **C** vs **D** was lower but still good, and **E** vs **F** was fair. ICC between **F** vs **A** or **B** was poor. *ICC* intraclass correlation

We considered SRIA in paraffin sections as the reference throughout the results. Median interstitial fibrosis scores according to the Remuzzi et al. (IF score) performed in H&E of frozen or in paraffin sections were both 2 (1–2) at original time. Weighted quadratic Cohen’s kappa for original IF scores in frozen vs paraffin sections was 0.47 corresponding to moderate agreement, whereas ICC for SRIA in frozen vs paraffin sections was 0.69 (95% CI 0.504–0.805 (F(72,72) = 3.212, p < 0.001, Fig. 4))), which is a good agreement. This indicated the good consistency of the computer-assisted evaluation, superior to the moderate agreement in optic microscopy.

Since we could not retrieve the original percentage of interstitial fibrosis detected by on-call pathologists who routinely provided the IF score only, we had to translate the computer-based measurements of interstitial fibrosis expressed in % of the cortical surface into the IF score, acknowledging a loss of granularity. Thereby, almost all samples showed lower IF scores in the original reports from an on-call pathologist than in our computerised analysis. IF scores based on SRIA in frozen sections vs paraffin sections had an ICC = 0.47 (95% CI 0.175–0.667 (F(72,72) = 1.949, p = 0.003))), which was a moderate agreement, whereas it was good with the computerised method. Comparing the IF score based on SRIA from frozen sections to the original FS report, there was a κ = 0.03, from paraffin section to the original report based on paraffin section, there was a κ = 0.04, and SRIA from paraffin sections vs the original frozen section reports a κ = 0.04, all three κ-values being poor (Fig. 4). So, translating the automated measurements into categorical values according to Remuzzi et al., resulting in a frank loss of agreement between frozen and paraffin sections, we were able to demonstrate the superior consistency and accuracy of the quantitative evaluation of IF by computerised analysis.

### No clinical factor influences the quantitative difference of interstitial fibrosis between frozen and paraffin sections

To identify factors influencing the variations of the measurements of interstitial fibrosis in frozen and paraffin sections, we sought to test the influence of donor age (absolute or over 60 years, as used otherwise [13, 37]), sex, CIT over 20 h, or NHBD using two independent methods.

We first blended these single factors into the Bland–Altman plot for the difference of SRIA in frozen vs paraffin sections. They did not modify the plot significantly, with variations of mean difference of SRIA between 7.5 and 9.4% for donor age, sex and CIT over 20 h or 5.5% for NHBD (all p-values > 0.05, see supplementary material S1). We then challenged these factors directly at the levels of quantitative and semi-quantitative evaluation. We defined relevant differences between measurements as a minimum of 10% variation between SRIAs or any difference between the original and the computer-derived IF scores and could not find any significant influencing factor on the variations either.

### SRIA in paraffin section predicts DGF

None of our estimation methods for interstitial fibrosis yielded a statistically significant value for the prediction of graft survival, rejection episodes or eGFR at 1, 3, 6 or 12 months (data not shown). Nonetheless, univariate analysis for risk factors of DGF (n = 29 (41%)) identified SRIA in paraffin section and cold ischaemia time (odds ratio (OR) = 1.11 (95% confidence interval (CI) 1.022–1.206); p = 0.014, intercept = − 0.37 and OR = 1.102 (95%CI 1.001–1.213); p = 0.048, intercept = − 0.36, respectively—see Table 2). Male donor gender reached a trend towards significance (OR = 2.596 (95%CI 0.913–7.384; p = 0.074, intercept = − 0.37)). In a multivariate analysis including SRIA in paraffin sections, CIT and male donor gender, SRIA was an independent significant risk factor for DGF (OR = 1.1 (95%CI 1.011–1.198; p = 0.028, intercept = − 0.36)). There was no co-linearity detectable (variance inflation factor: 1.042–1.069). Models including SRIA in the frozen section, the original or the calculated IF scores did not yield comparable results (Supplementary material S2).

**Table 2:** Uni- and multivariate logistic regression analyses of predictors for delayed graft function (DGF)

	Univariate analysis			Multivariate analysis		
	Odds ratio	95% CI	*p* value	Odds ratio	95% CI	*p* value
SRIA PS in %	1.11	1.022–1.21	**0.014**	1.1	1.011–1.198	**0.028**
SRIA FS in %	1.017	0.943–1.1	0.659			
IF ac. Remuzzi	0.721	0.285–1.82	0.489			
Final grade ac. Remuzzi	1.495	0.539–4.15	0.44			
CIT in hours	1.102	1.001–1.21	**0.048**	1.089	0.981–1.21	0.111
Donor age in years	1.003	0.965–1.4	0.862			
Male donor	2.596	0.913–7.394	0.074	1.99	0.64–6.17	0.231
Recipient age in years	1.01	0.967–1.05	0.642			
Male recipient	0.413	0.556–4.1	0.418			
DSA presence	0.667	0.121–3.7	0.641			

Significant values are in bold

SRIA in paraffin section and cold ischaemia time significantly predict DGF in univariate analysis, and male donor gender reaches a trend (p < 0.1). In multivariate analysis, SRIA in paraffin section is an independent predictor of DGF

*CI* confidence interval, *CIT* cold ischaemia time, *DSA* donor-specific antibody, *IF ac. Remuzzi* interstitial fibrosis grade according to Remuzzi et al., *Final grade ac. Remuzzi* Final grade according to Remuzzi et al., *SRIA FS* Sirius red-stained interstitial area in frozen section, *SRIA PS* Sirius red-stained interstitial area in paraffin section

## Discussion

Results of implantation biopsies, based on the evaluation of frozen sections, must provide sound information on the quality of tissue or existing diseases, as they are often the first reference in deciding whether or not to engraft an organ [9]. Interstitial fibrosis, an essential factor in the assessment of organ quality [38], is, however, problematic to quantify in frozen sections [14, 28]. We hypothesised that frozen sections might exaggerate interstitial collagenous content compared to the gold standard of paraffin sections, and sought to quantify this variation by digital image analysis. We then tested whether DIA of interstitial fibrosis might enhance the informative value of implantation biopsy specimens.

In our study, the Sirius red-stained interstitial area measured by DIA in the percentage of the surface of the same biopsy sample was greater in frozen than in paraffin sections. We identified a mean overestimation of 7.9% for frozen sections, with variations of up to 29% in single cases. A Bland–Altman plot showed that SRIA in a frozen section compared to a paraffin section does not suffer from proportional bias. This meant that kidney specimens with growing interstitial fibrosis display a rather constant, but not gradually stronger, augmentation of interstitial fibrosis in a frozen section compared to a paraffin section. Donor-related factors such as age, gender, NHBD, and cold ischaemia time did not influence this difference significantly. As the thickness between the section groups could have played a substantial role in the detection of the interstitial volume, we sought to systematically prepare sections of the same thickness (4 μm) for both frozen and paraffin sections. Moreover, only one technician supervised the staining of the “section pairs”, performed synchronously. Hence, the histological technique of frozen section by itself appears to be the main reason for the apparent augmentation of interstitial fibrosis. We could now provide precise quantification of this difference. Consequently, a cautious evaluation of interstitial fibrosis is warranted and pathologists should refrain from overrating this feature in frozen sections.

One way to enhance the quality and reproducibility of reports on implantation biopsies is the routine application of consensual scoring systems. The most broadly used assessment system is the classification by Remuzzi et al., initially applied in the context of extended donor criteria, and utilised in our centre for marginal and standard donor kidneys that are clinically or macroscopically suspicious [25]. We, on the other hand, assumed that a quantitative assessment supported by DIA would outperform this method, and, hence, addressed the consistency of our computerised approach compared to the original IF score according to Remuzzi et al. made in frozen and paraffin sections. Evaluating the differences between a semi-quantitative and a quantitative method often provides varying values for the same interstitial area, with a good correlation for mild fibrosis but a poor correlation for severe fibrosis [23]. Our study population included many cases with moderate to severe interstitial fibrosis, and we could verify this former observation, with an overall need to upgrade the original frozen sections assessment and lower intraclass correlation values. So, in our study, on-call pathologists tended to underestimate interstitial fibrosis in frozen sections, contradicting our first concerns. Sagasta et al., from another European centre, described a similar phenomenon between on-call and definitive reports: in a retrospective analysis, their nephropathologists systematically upgraded the original scores [39]. In our cohort, the ICC for interstitial fibrosis between the original report made in frozen sections and the definitive report made in paraffin sections was good, but the ICC of the computerised quantitative measurements was superior. In contrast, when we translated our quantitative measurements into semi-quantitative IF scores, it lowered the ICC. Subsequent comparison of the performance between the calculated IF scores from the computerised measurements (our reference) vs. the original IF scores showed even poorer ICC. So, the optical microscopy evaluation was less consistent. Another study, by Liapis et al. pointed out the poor reproducibility of the semi-quantitative evaluation method in optical microscopy of implantation biopsies, with very low ICC ranging from -0.013 for frozen to 0.044 for paraffin sections of core biopsies [13]. Our higher ICC must be considered in view of the fact that the setting of this earlier study was different: 32 pathologists participated, the evaluation was based on scanned H&E but not collagen-specific Sirius red-stained sections, and they applied thresholds of the Banff classification (< 5%, 6–25%, 26–50% and > 50%). However, with an ICC of 0.69, our method represents a significant improvement in this regard.

We could not identify a predictive value of the interstitial fibrosis quantification either with the SRIA in frozen sections or with the semi-quantitative methods. However, the quantification of interstitial fibrosis computerised as SRIA from paraffin sections showed to be significantly predictive of a relevant clinical situation, i.e., delayed graft function. DGF is a risk factor for the occurrence of rejection episodes at one year and poorer renal allograft outcome short- or long-term [40–42]. An earlier study stated that ci score > 0 according to the Banff classification in reperfusion biopsies was not significantly associated with DGF [43]. This mere dichotomous separation for the presence or absence of interstitial fibrosis is, in our view, probably not adequate to capture the complexity of the lesion [9]. Our results, furthermore, complete the earlier work by Liapis et al., showing in their smaller data set that intermediate-grade interstitial fibrosis reaches a trend for the prediction of DGF [13]. A high SRIA in paraffin sections is, therefore, relevant and could affect post-operative and long-term management [41].

A possible drawback of our work is the high sensitivity of our approach, which is due to the accurate computer-based measurements and the use of the collagen-specific reference stain Sirius Red. Indeed, routine studies are based on evaluation by light microscopy and H&E in frozen sections, which may compromise a more precise quantitative analysis. Sirius red staining has, however, already been proposed to study interstitial fibrosis in renal allograft biopsies [27]. However, the median of our reference measurement was comparable to some published data (32% vs mean of 31% [44]); and different from others (32% vs 8.4% [31] or vs 9.5% [23]), which points to varying baseline measurements of interstitial fibrosis in various patient cohorts. It is, moreover, worth reiterating that our study specimens had been gathered based on morphological or clinical indication, which implies candidate kidneys with more chronic damage. Besides, when comparing our method to the performance of the semi-quantitative approach, we could hardly include a “zero” category for IF score, defined in the original work of Remuzzi et al. as “absent” [25], which can be broadly interpreted in the practice, but not by our computer (even 0.001% of fibrosis is not zero for the computer). An inherent bias of our approach lies in the fact that we compared two adjacent sections, understanding that slight variations of the interstitial volume are basically present. However, operator-based exclusion of glomerular or arterial structures and the overall “buffering” of the changes in interstitial volume in a total of 73 section pairs (i.e. in some pairs more IF differences and in other pairs less IF differences) compensate for these inevitable variations. All kidneys included in our study were implanted, hence not allowing us to assess the effect of findings of interstitial fibrosis on discard rates. We, furthermore, cannot exclude the possibility that the retrospective character of our study with some missing data has impeded the detection of other predictive factors.

So, paradoxically, while on-call pathologists tend to underestimate renal interstitial fibrosis in frozen sections, the frozen section technique itself artificially amplifies the perception of interstitial fibrosis, as compared to the gold standard of paraffin section. This is, to our knowledge, the first publication precisely quantifying this difference by DIA [21]. Hence, we recommend a cautious evaluation of the interstitial fibrosis in frozen sections, refraining from using too loose thresholds and well acknowledging that the frozen section technique may artificially exaggerate the interstitial matrix. For the evaluation of interstitial fibrosis, our results confirm the superiority of paraffin over frozen sections, and of DIA over routine optical microscopy. Conversely, one could claim that frozen sections processed by on-call pathologists allow a quick report satisfactory enough for the practice. We believe that the rapid processing of samples and an efficient DIA could compensate for the rapidity of frozen sections. Previous efforts for the implementation of digital pathology in the evaluation of implantation biopsies support this view, as it enhanced precision and allowed better reproducibility [20]. Consequently, the extension of DIA to rapidly processed implantation biopsies should be tested. In our opinion, evaluation of other histological features in kidneys or even in other organs is certainly worth encouraging. In addition, we also advocate the evaluation of renal interstitium in kidneys with lesser tissue damage. While developing a user-friendly and time-saving system will be one of the significant challenges before its broad implementation, DIA seems to be a robust method in comparison to the current procedures. A more accurate morphological appraisal of implantation biopsies would ultimately spare organs currently discarded based on pathology reports, and help to reduce long waiting lists for transplantation.

## Supplementary Information

Below is the link to the electronic supplementary material.

Supplementary file1 (PDF 441 KB)

Supplementary file2 (PDF 56 KB)

## Abbreviations

BDD: Brain-dead donor
CIT: Cold ischaemia time
CKD EPI: Chronic kidney disease epidemiology
DGF: Delayed graft function
DIA: Digital image analysis
DSA: Donor-specific antibody
eGFR: Estimated glomerular filtration rate
ESRD: End-stage renal disease
H&E: Haematoxylin and eosin
HLA: Human leukocyte antigen
NHBD: Non-heart-beating donor
ICC: Intraclass correlation
IF: Interstitial fibrosis
IQR: Interquartile range
PRA: Panel-reactive antibody
SD: Standard deviation
SRIA: Sirius red-stained interstitial area
