# Supplementary material for: Computer-assisted evaluation enhances the quantification of interstitial fibrosis in renal implantation biopsies, measures differences between frozen and paraffin sections, and predicts delayed graft function
Source: J Nephrol. 2022 Apr 19;35(7):1819–29. doi: 10.1007/s40620-022-01315-y (PMC9458593; doi:10.1007/s40620-022-01315-y)
Supplement: Supplementary file 1 — Supplementary file1 (PDF 441 KB) [file 40620_2022_1315_MOESM1_ESM.pdf]

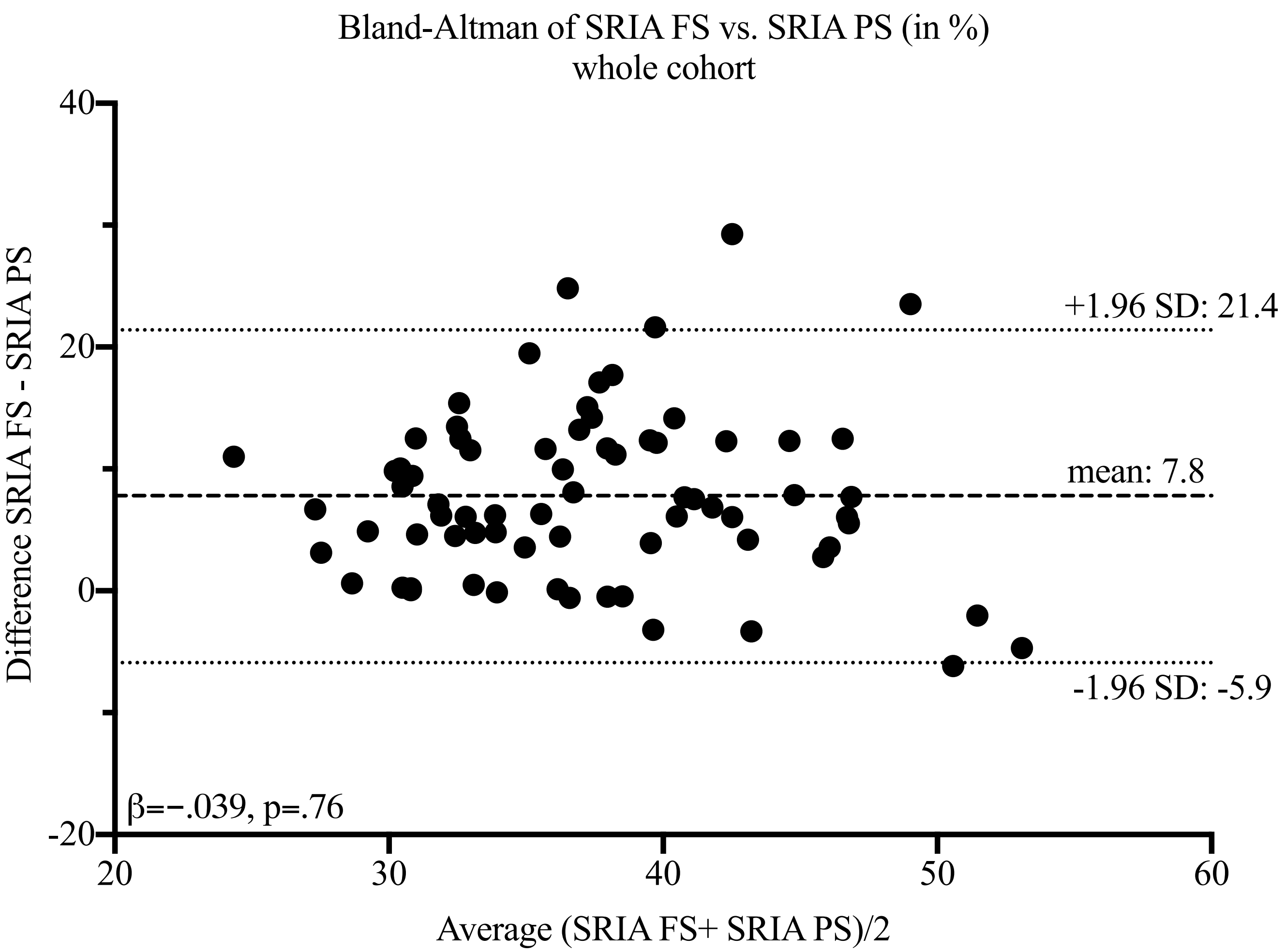

Bland-Altman of SRIA FS vs. SRIA PS (in %)
whole cohort
40
20
0
-20
Difference SRIA FS - SRIA PS
+1.96 SD: 21.4
mean: 7.8
-1.96 SD: -5.9
β=−.039, p=.76
20
30
40
50
60
Average (SRIA FS+ SRIA PS)/2

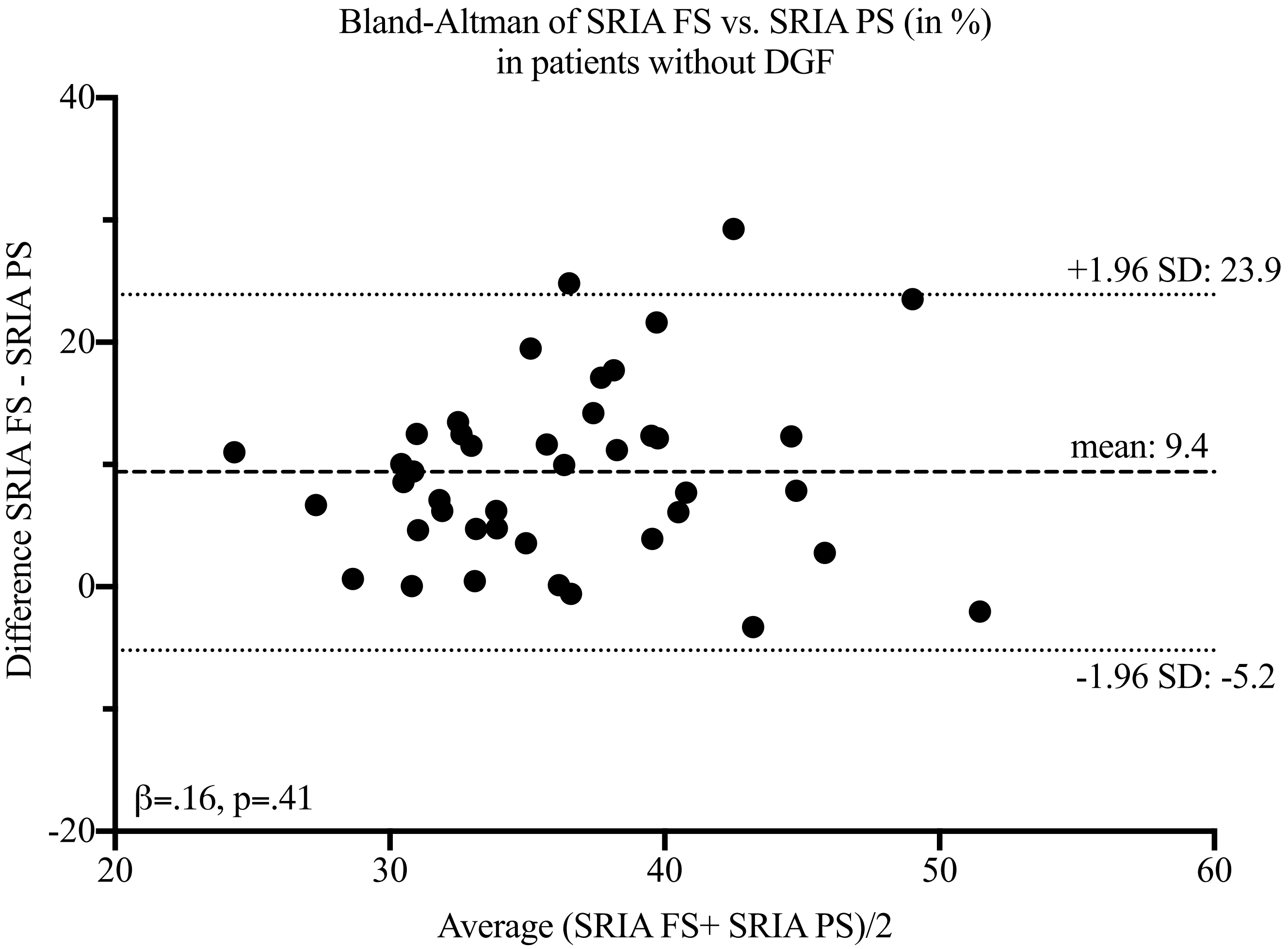

Bland-Altman of SRIA FS vs. SRIA PS (in %)
in patients without DGF
Difference SRIA FS - SRIA PS
Average (SRIA FS+ SRIA PS)/2
+1.96 SD: 23.9
mean: 9.4
-1.96 SD: -5.2
β=.16, p=.41
40
20
0
-20
20
30
40
50
60

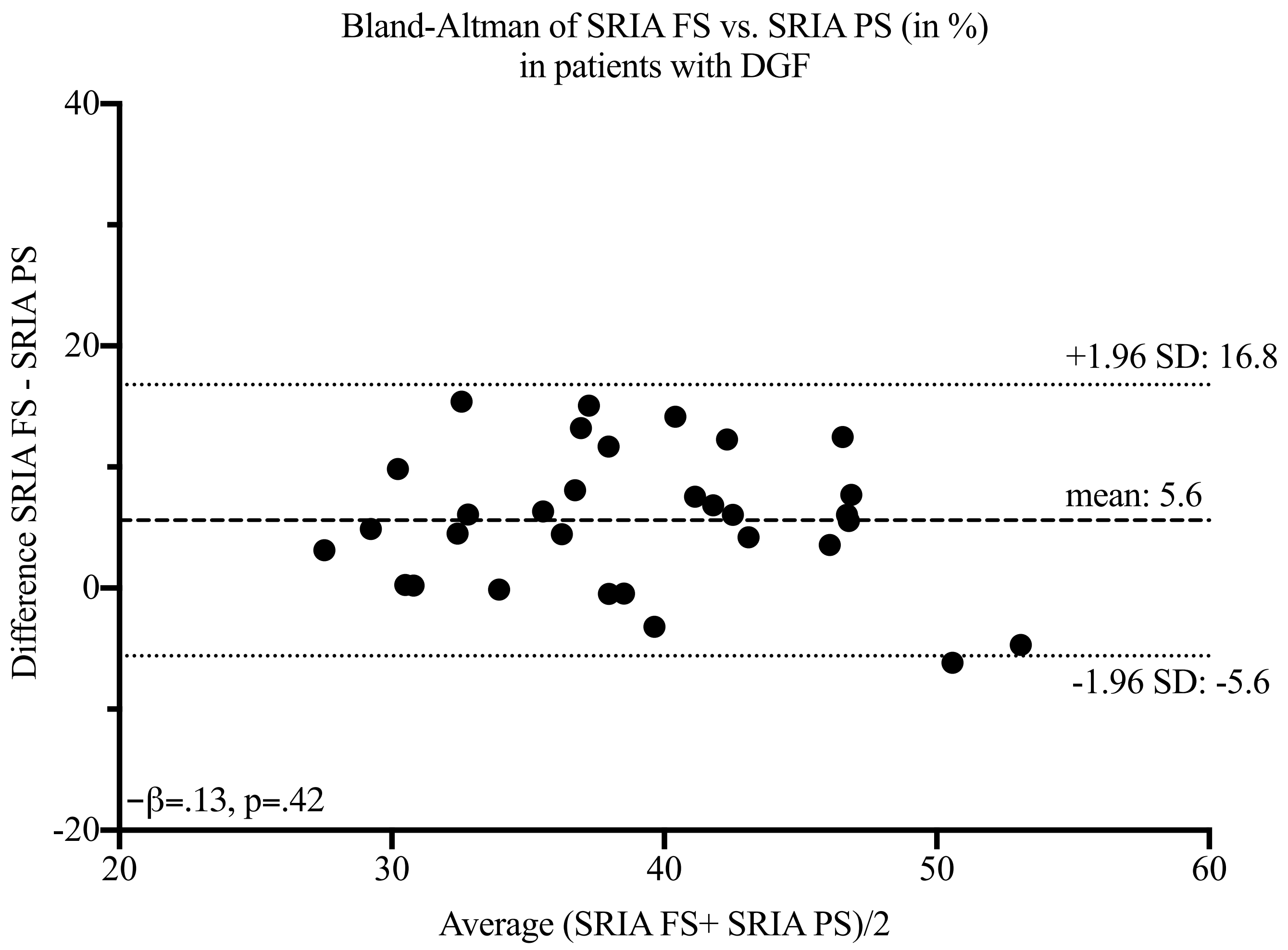
Bland-Altman of SRIA FS vs. SRIA PS (in %)
in patients with DGF
40
20
0
-20
20
30
40
50
60
+1.96 SD: 16.8
mean: 5.6
-1.96 SD: -5.6
−β=.13, p=.42
Difference SRIA FS - SRIA PS
Average (SRIA FS+ SRIA PS)/2

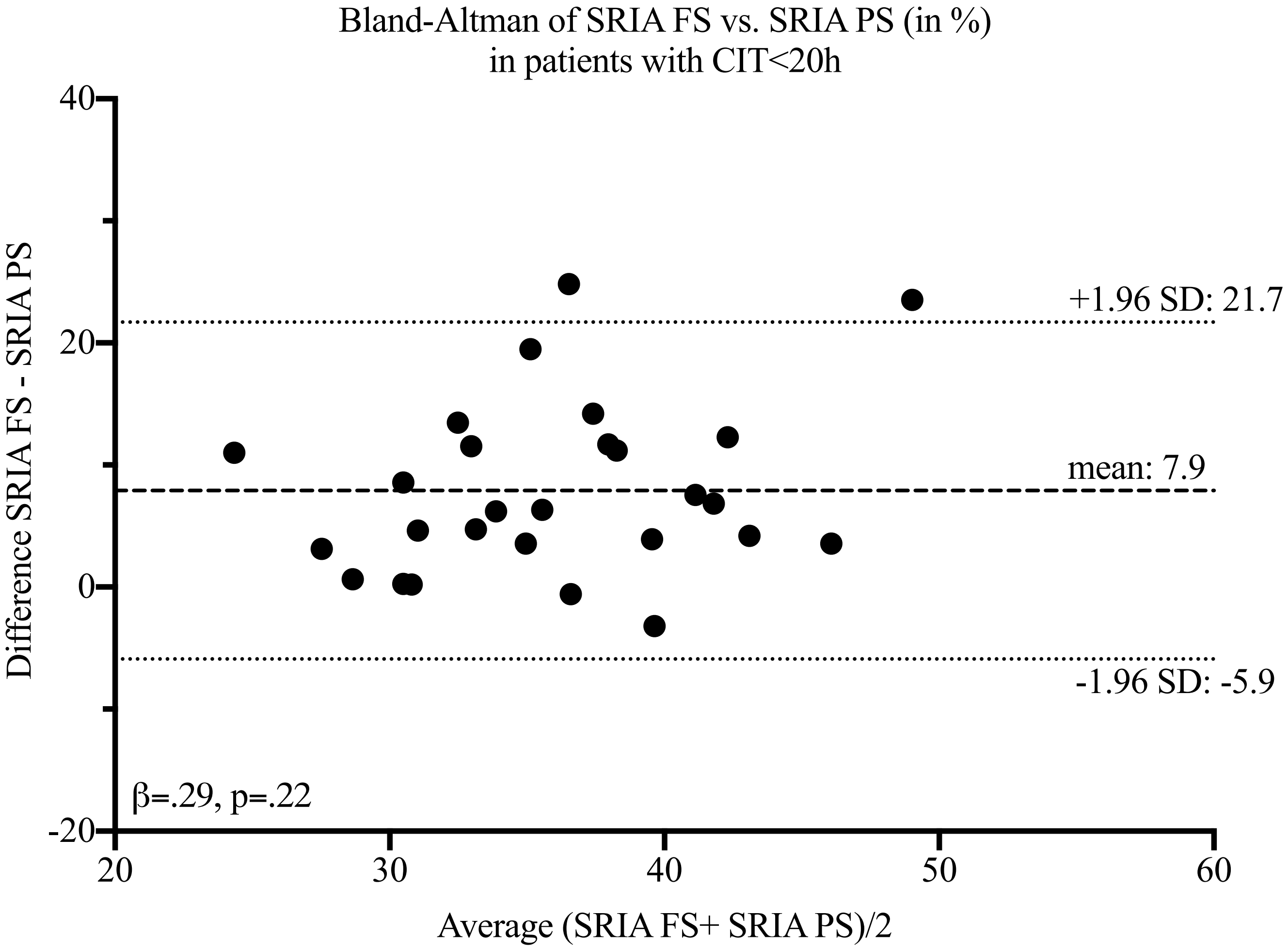

Bland-Altman of SRIA FS vs. SRIA PS (in %)
in patients with CIT<20h
Difference SRIA FS - SRIA PS
Average (SRIA FS+ SRIA PS)/2
+1.96 SD: 21.7
mean: 7.9
-1.96 SD: -5.9
β=.29, p=.22
40
20
0
-20
20
30
40
50
60

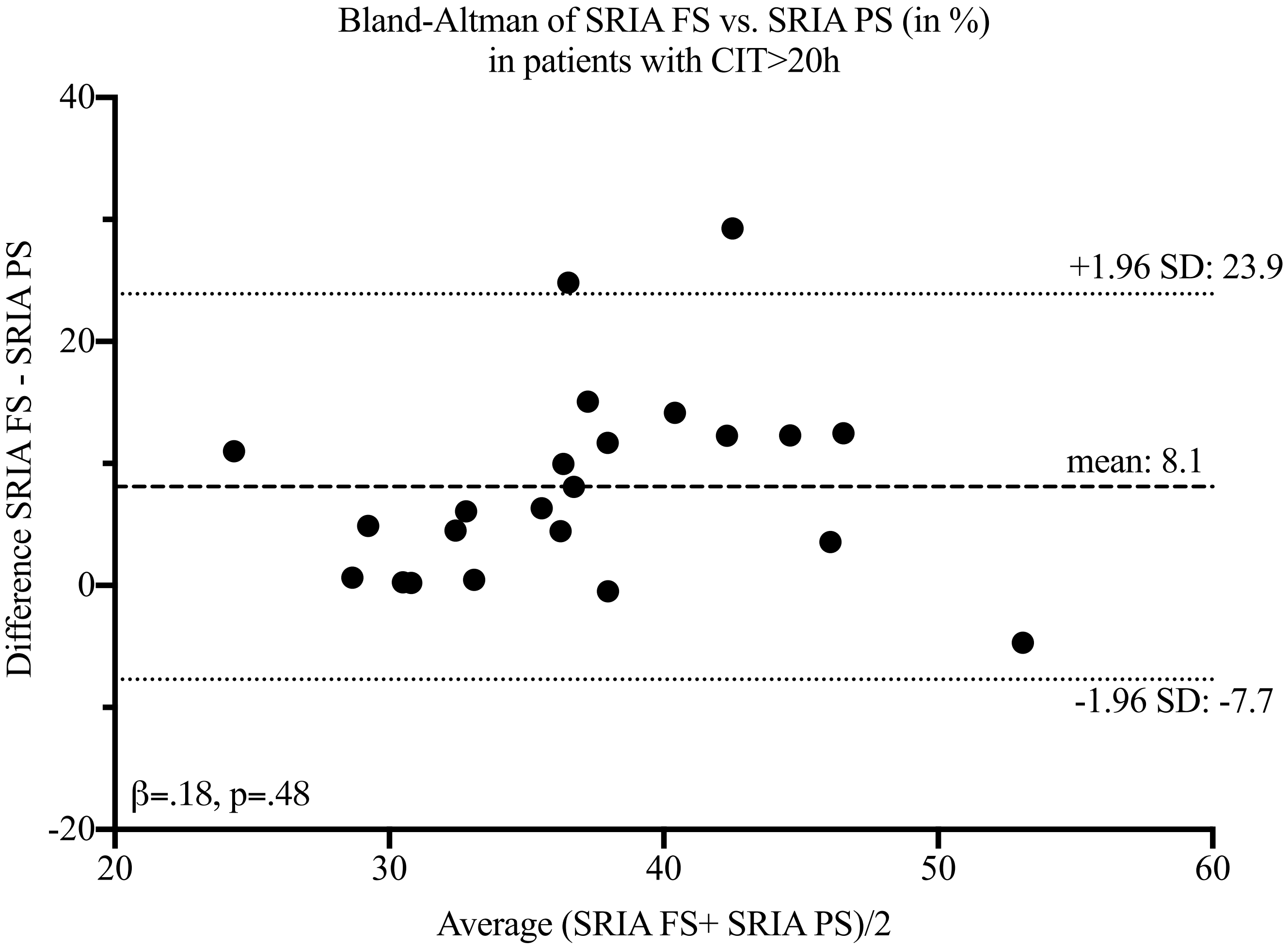

Bland-Altman of SRIA FS vs. SRIA PS (in %)
in patients with CIT>20h
Difference SRIA FS - SRIA PS
Average (SRIA FS+ SRIA PS)/2
+1.96 SD: 23.9
mean: 8.1
-1.96 SD: -7.7
β=.18, p=.48
40
20
0
-20
20
30
40
50
60

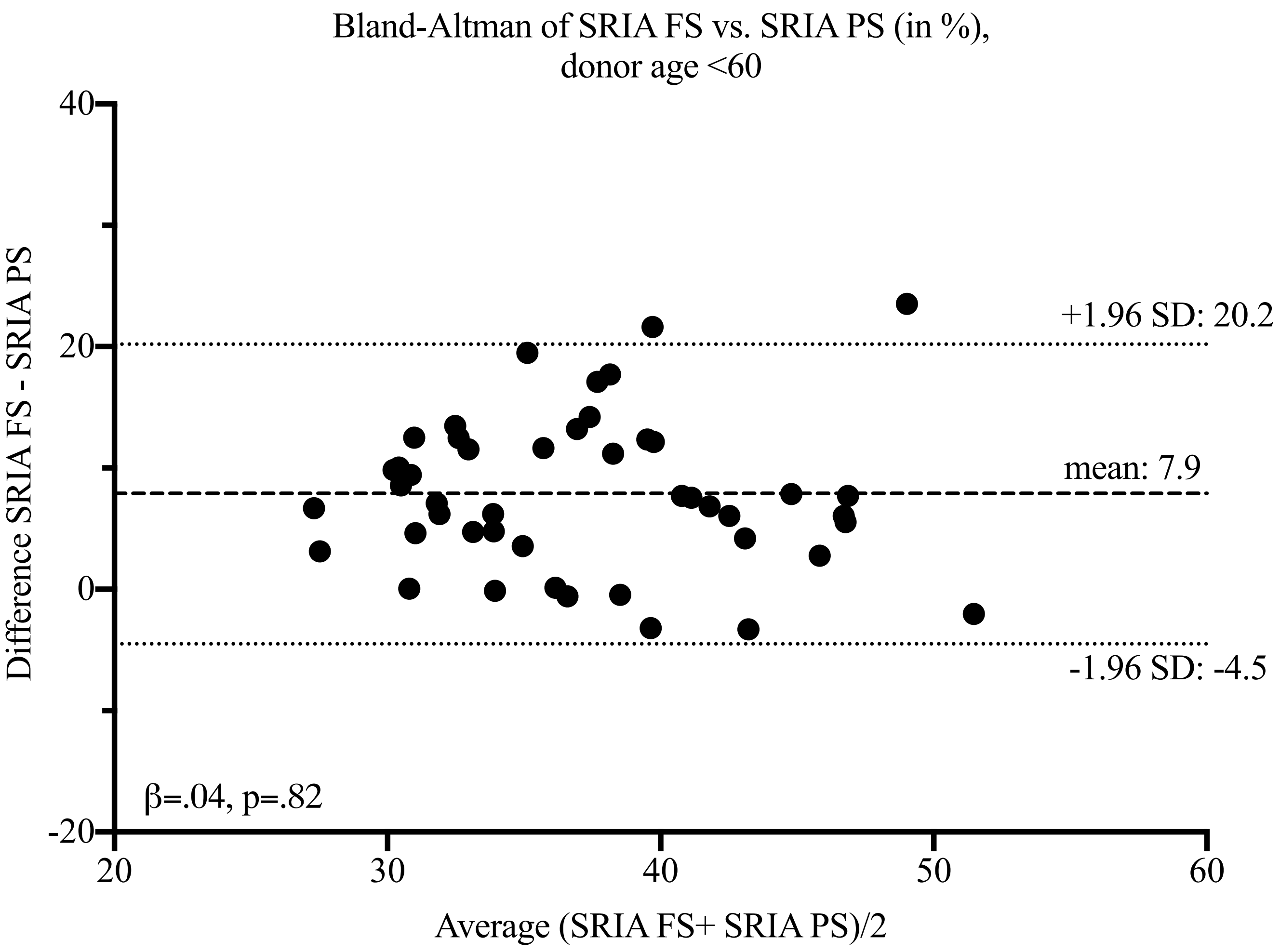

Bland-Altman of SRIA FS vs. SRIA PS (in %),
donor age <60
Difference SRIA FS - SRIA PS
Average (SRIA FS+ SRIA PS)/2
+1.96 SD: 20.2
mean: 7.9
-1.96 SD: -4.5
β=.04, p=.82
40
20
0
-20
20
30
40
50
60

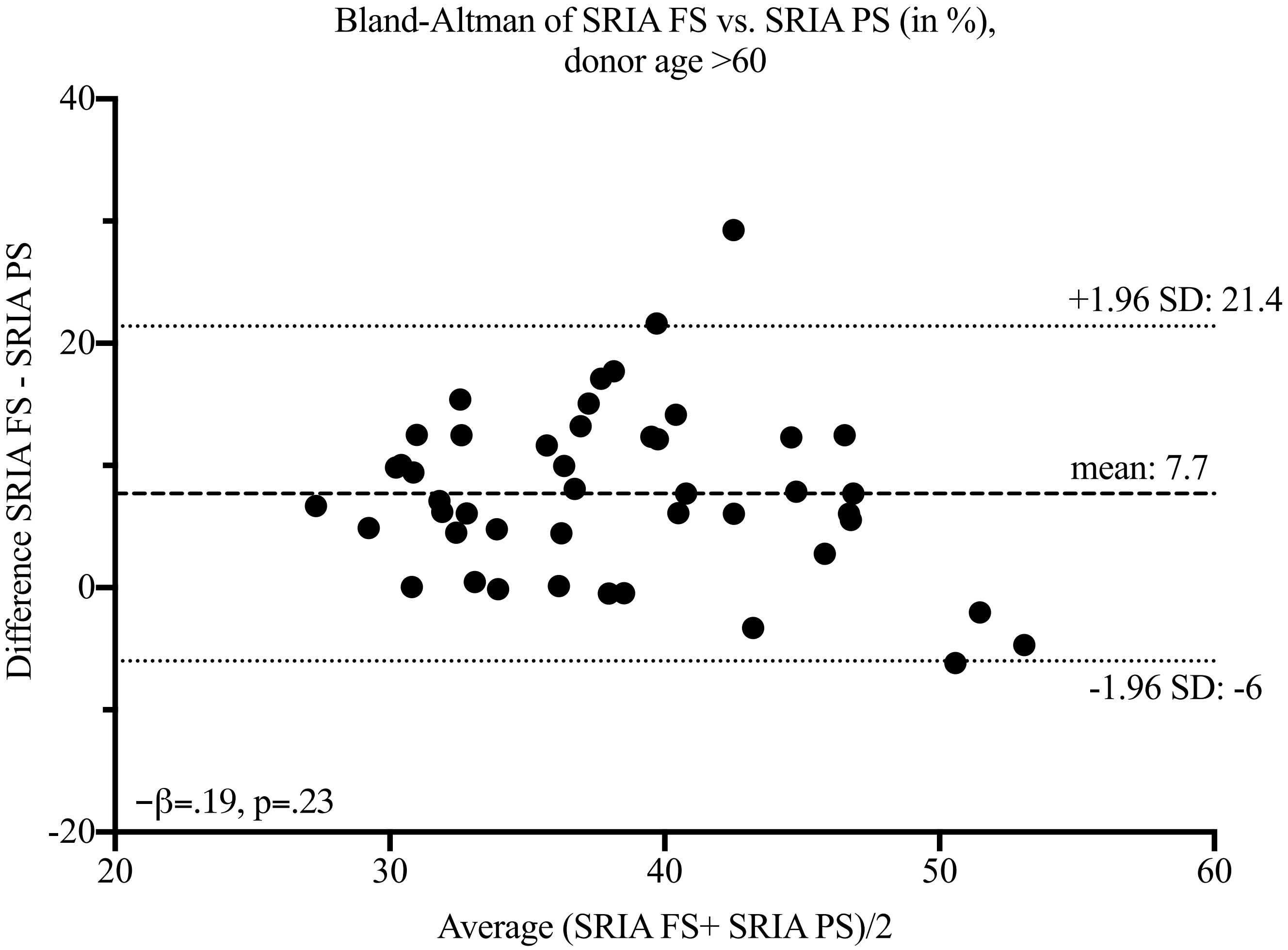
Bland-Altman of SRIA FS vs. SRIA PS (in %),
donor age >60
Difference SRIA FS - SRIA PS
Average (SRIA FS+ SRIA PS)/2
+1.96 SD: 21.4
mean: 7.7
-1.96 SD: -6
−β=.19, p=.23
40
20
0
-20
20
30
40
50
60

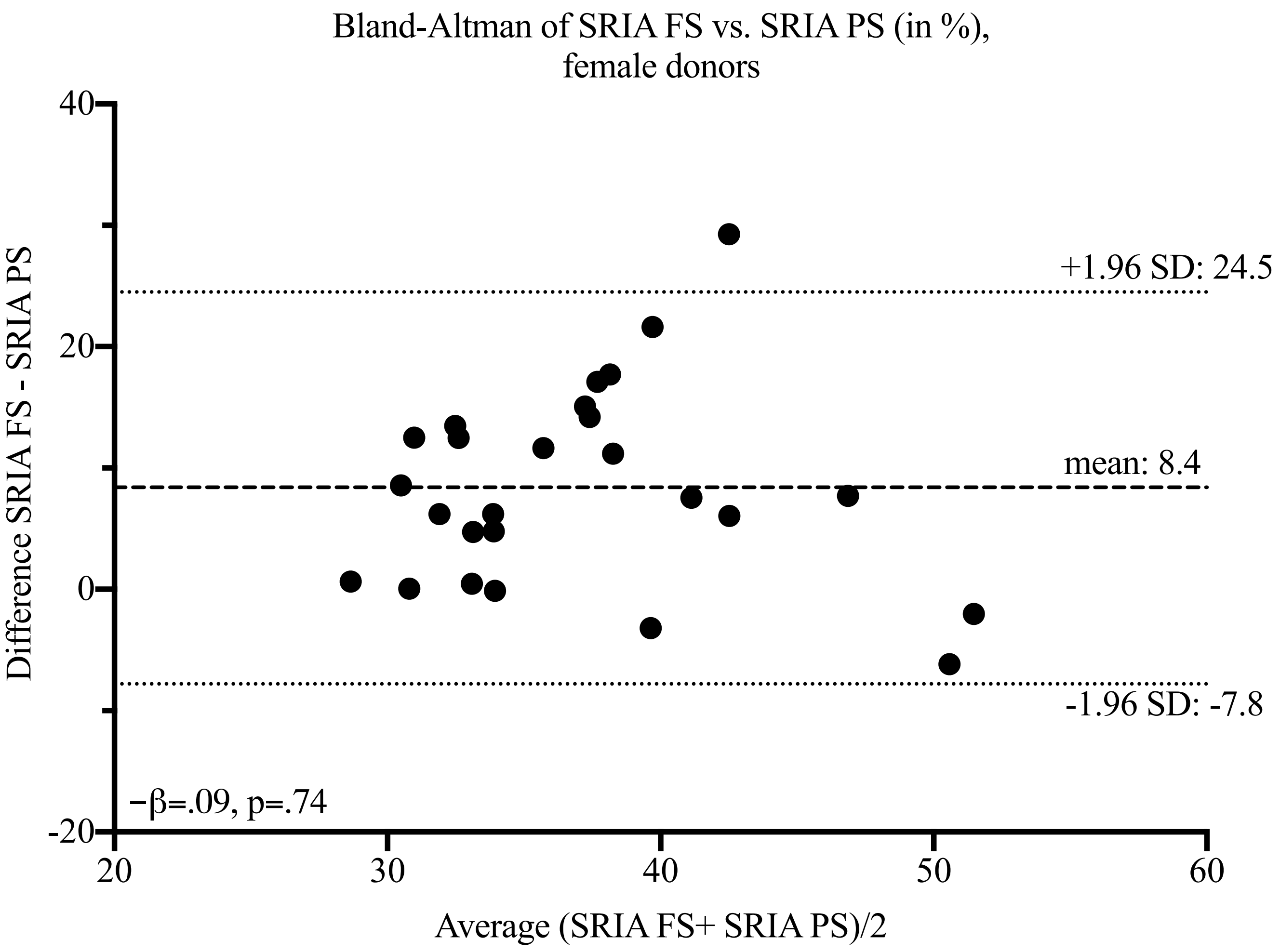

Bland-Altman of SRIA FS vs. SRIA PS (in %),
female donors
Difference SRIA FS - SRIA PS
Average (SRIA FS+ SRIA PS)/2
+1.96 SD: 24.5
mean: 8.4
-1.96 SD: -7.8
−β=.09, p=.74
40
20
0
-20
20
30
40
50
60

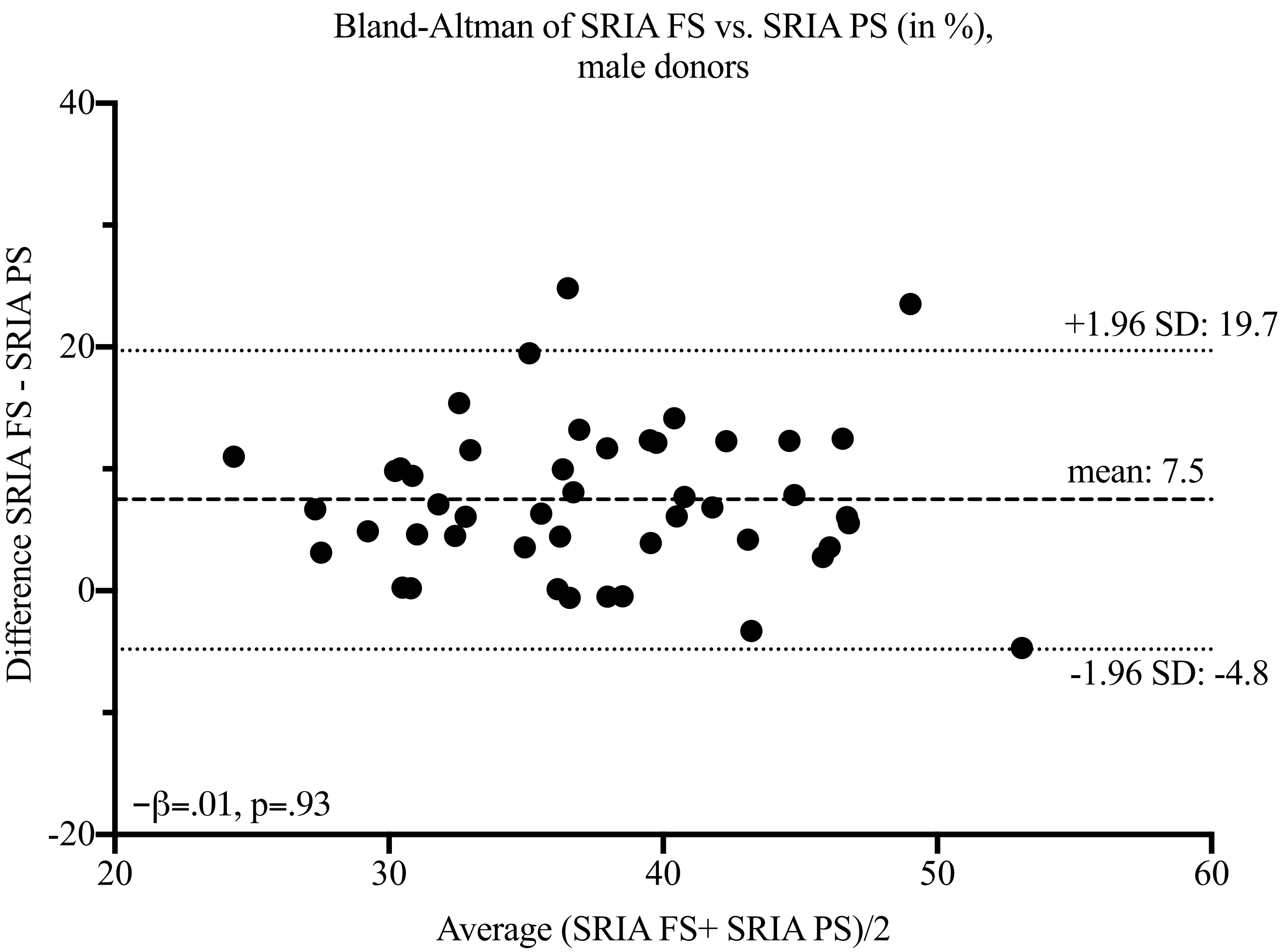

Bland-Altman of SRIA FS vs. SRIA PS (in %),
male donors
Difference SRIA FS - SRIA PS
Average (SRIA FS+ SRIA PS)/2
+1.96 SD: 19.7
mean: 7.5
-1.96 SD: -4.8
−β=.01, p=.93
40
20
0
-20
20
30
40
50
60

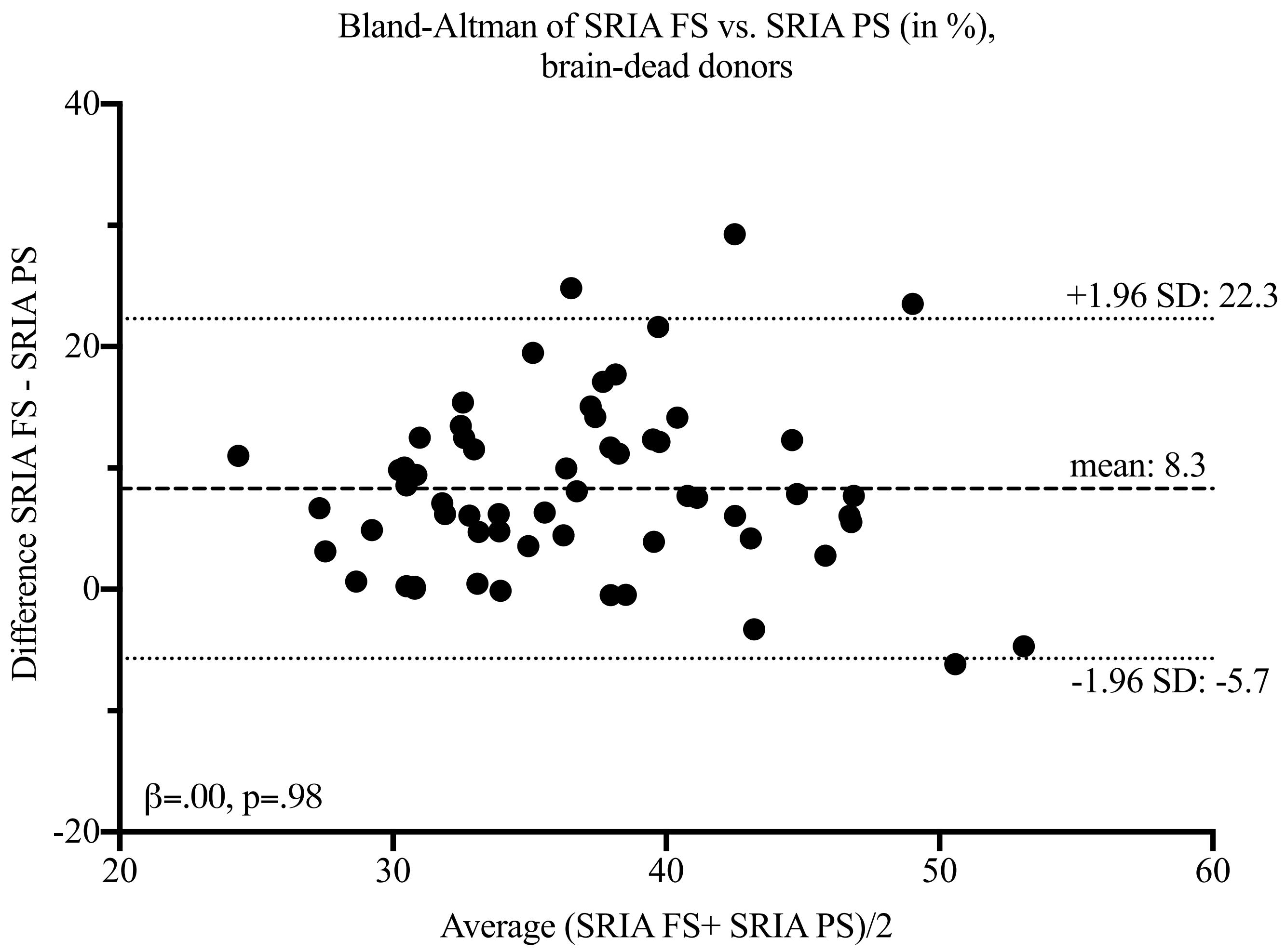
Bland-Altman of SRIA FS vs. SRIA PS (in %),
brain-dead donors
Difference SRIA FS - SRIA PS
Average (SRIA FS+ SRIA PS)/2
+1.96 SD: 22.3
mean: 8.3
-1.96 SD: -5.7
β=.00, p=.98
40
20
0
-20
20
30
40
50
60

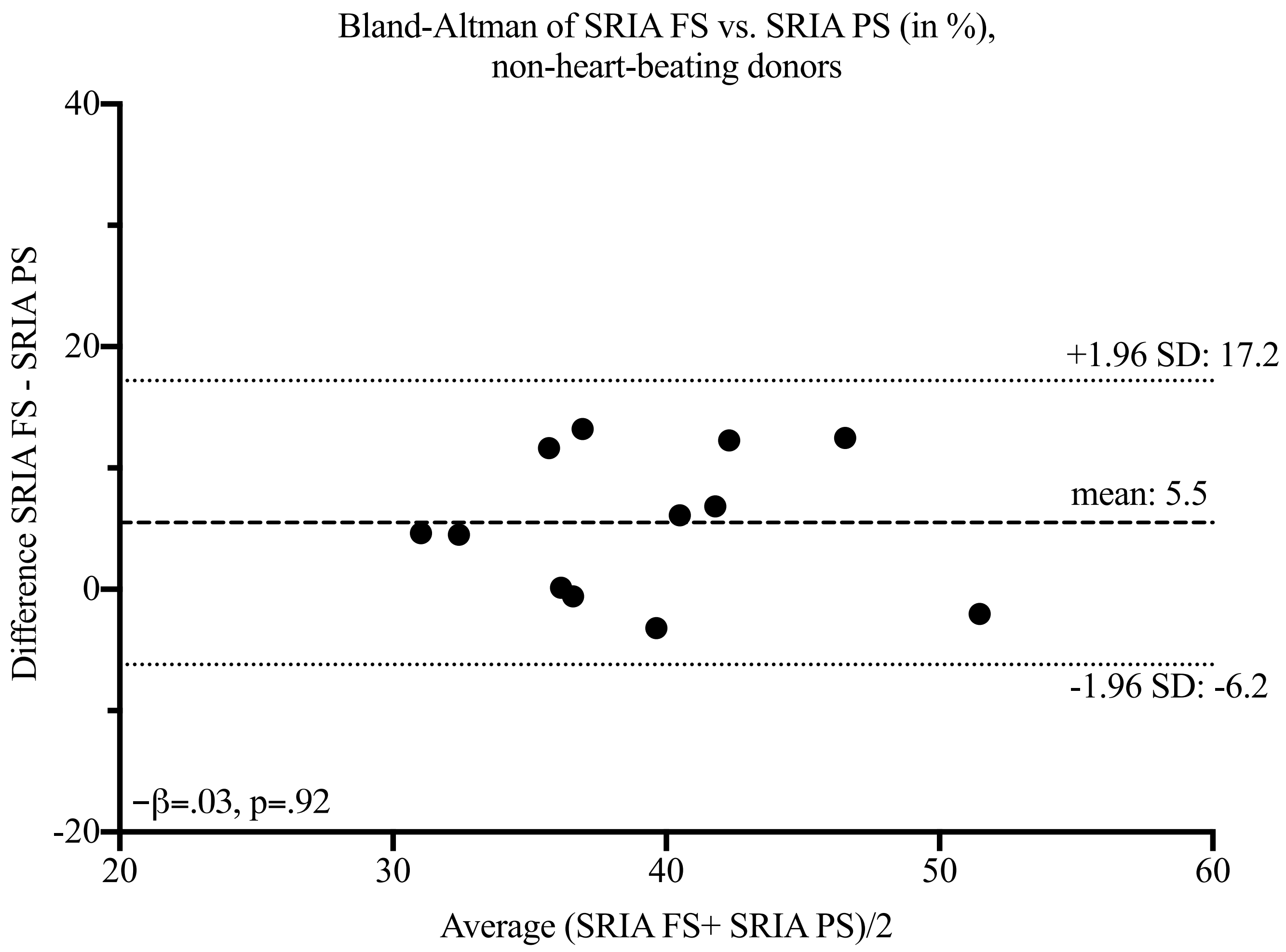
Bland-Altman of SRIA FS vs. SRIA PS (in %),
non-heart-beating donors
Difference SRIA FS - SRIA PS
Average (SRIA FS+ SRIA PS)/2
+1.96 SD: 17.2
mean: 5.5
-1.96 SD: -6.2
−β=.03, p=.92
40
20
0
-20
20
30
40
50
60
