# Supplementary material for: Computer-assisted evaluation enhances the quantification of interstitial fibrosis in renal implantation biopsies, measures differences between frozen and paraffin sections, and predicts delayed graft function
Source: J Nephrol. 2022 Apr 19;35(7):1819–29. doi: 10.1007/s40620-022-01315-y (PMC9458593; doi:10.1007/s40620-022-01315-y)
Supplement: Supplementary file 2 — Supplementary file2 (PDF 56 KB) [file 40620_2022_1315_MOESM2_ESM.pdf]

	Multivariate analysis			Multivariate analysis			Multivariate analysis			Multivariate analysis		
	Odds ratio	95% CI	p-value	Odds ratio	95% CI	p-value	Odds ratio	95% CI	p-value	Odds ratio	95% CI	p-value
SRIA FS in %	1.01	.94-1.1	.85									
IF RS in FS				.79	.48-2.6	.79						
SRIA FS in IF RS							.55	.11-2.7	.47			
SRIA PS in IF RS										2.7	.2-27	.49
CIT in hours	1.09	.99-1.2	.084	1.1	.99-1.2	.066	1.1	.99-1.2	.066	1.09	.99-1.2	.074
Male donor	2.4	.81-7.1	.112	2.48	.84-7.4	.101	2.48	.84-2.7	.101	2.36	.79-7	.12

Multivariate logistic regression analyses of predictors for delayed graft function. None of the other measurement methods of interstitial fibrosis could predict DGF as did SRIA PS in %. CI: confidence interval; CIT: cold ischaemia time; DSA: donor-specific antibody; FS: frozen section; IF RS: interstitial fibrosis grade according to Remuzzi et al; PS: paraffin section; SRIA FS, Sirius red-stained interstitial area in frozen section; SRIA PS, Sirius red-stained interstitial area in paraffin section.
